# Diagnostic Assessment of Adult Hydrocephalus Log compared to standard normal pressure hydrocephalus diagnostic tools

**DOI:** 10.1186/2045-8118-12-S1-O44

**Published:** 2015-09-18

**Authors:** Ignacio Jusue-Torres, Jennifer Lu, Jamie Robison, Jamie Hoffberger, Jan Wemmer, Abanti Sanyal, Daniele Rigamonti

**Affiliations:** 1 Johns Hopkins University, School of Medicine, Department of Neurosurgery, USA; 2 Johns Hopkins University, Bloomberg School of Public Health, Department of Biostatistics, USA

## Introduction

Early treatment in Normal Pressure Hydrocephalus (NPH) yields better post-operative outcomes. Our current tests often fail to detect significant changes at early stages. We developed a new scoring system (LP log score) and sought a “proof of concept” that this tool is more sensitive in detecting clinical differences than the current ones.

## Methods

We prospectively studied 62 consecutive new patients with suspected idiopathic NPH. Secondary, previously treated and obstructive cases were not included. We collected age, pre and post Lumbar Puncture (LP) Tinetti, Timed Up and Go (TUG), European NPH scale and LP log scores. LP log score is recorded at baseline and for 7 consecutive days after removing 40 cc of CSF via LP. We studied the diagnostic accuracy of the tests for surgical indication.

## Results

Median age at presentation was 76 (71-80) years old. TUG (p<0.0001) and Tinetti (p<0.0001) showed significant differences between presentation and post-LP scores. PostLP Log showed improvement in 90% of people with good baseline TUG, Tinetti and MCV tests and in 93% of people who did not show any pre-post LP change in TUG, Tinetti and MCV grade. Sensitivity, Specificity, and Accuracy to detect intention to treat when positive postLP improvements were respectively 4%, 100% and 24% for TUG, 21%, 86%, 34% for Tinetti, 66%, 29% and 58% for MCV grade and 98%, 33% and 85% for LP log. PreLP-postLP TUG improvement and preLP-postLP Tinetti improvement were not associated with surgical indication (p>0.05). LP log improvement was associated with surgical indication OR: 24.5 95%CI (2.4-248.12) (p=0.007).

## Conclusions

LP log showed a higher sensitivity and diagnostic accuracy detecting clinical differences in NPH than the current diagnostic approach. Our next step is to conduct a cross-validation analysis of the diagnostic and prognostic accuracy of this new tool.

## References

[B1] Sankey EW Jusue-Torres I Elder BD Functional gait outcomes for idiopathic normal pressure hydrocephalus after primary endoscopic third ventriculostomy J Clin Neurosci 2015 1 6 25979256 10.1016/j.jocn.2015.02.019

[B2] Moran D Kosztowski TA Jusue-Torres I Does CT wand guidance improve shunt placement in patients with hydrocephalus? Clin Neurol Neurosurg 2015 132 26 30 25746318 10.1016/j.clineuro.2015.02.007

[B3] Jusue I Torres Hoffberger JB Rigamonti D Complications Specific to Lumboperitoneal Shunt. Complications of CSF Shunting in Hydrocephalus 2015 Springer 203 11

[B4] Elder BD Sankey EW Goodwin CR Jusue-Torres I Khattab MH Rigamonti D Outcomes and Experience with Lumbopleural Shunts in the Management of Idiopathic Intracranial Hypertension World Neurosurg 2015 1 6 10.1016/j.wneu.2015.03.021 25805534

[B5] Jusué-Torres I Hoffberger JB Rigamonti D Complications of Lumboperitoneal Shunts for Idiopathic Intracranial Hypertension Cureus 2014

[B6] Jusue I Torres Hoffberger JB Rigamonti D Complications of Lumboperitoneal Shunts for Normal Pressure Hydrocephalus Cureus 2014

[B7] Elder BD Bankah P Blitz AM Rigamonti D Core imaging in adult hydrocephalus 2014 Adult Hydrocephalus: Cambridge University Press 110 20

